# Finding the match between healthcare worker and expert for optimal audit and feedback on antimicrobial resistance prevention measures

**DOI:** 10.1186/s13756-020-00794-7

**Published:** 2020-08-05

**Authors:** J. Keizer, N. Beerlage-De Jong, N. Al Naiemi, J. E. W. C. van Gemert-Pijnen

**Affiliations:** 1 Centre for eHealth and Wellbeing Research, Department of Psychology, Health and Technology, University of Twente, P.O. Box 217, Enschede, 7500AE The Netherlands; 2 Department of Infection Prevention, Hospital Group Twente, Almelo/Hengelo, The Netherlands; 3 LabMicTA, Hengelo, The Netherlands

**Keywords:** Audit and feedback, Antimicrobial resistance, Healthcare worker, Participatory development

## Abstract

**Background:**

The potentials of audit and feedback (AF) to improve healthcare are currently not exploited. To unlock the potentials of AF, this study focused on the process of making sense of audit data and translating data into actionable feedback by studying a specific AF-case: limiting antimicrobial resistance (AMR). This was done via audit and feedback of AMR prevention measures (APM) that are executed by healthcare workers (HCW) in their day-to-day contact with patients. This study’s aim was to counterbalance the current predominantly top-down, expert-driven audit and feedback approach for APM, with needs and expectations of HCW.

**Methods:**

Qualitative semi-structured interviews were held with sixteen HCW (i.e. physicians, residents and nurses) from high-risk AMR departments at a regional hospital in The Netherlands. Deductive coding was succeeded by open and axial coding to establish main codes, subcodes and variations within codes.

**Results:**

HCW demand insights from audits into all facets of APM in their working routines (i.e. diagnostics, treatment and infection control), preferably in the form of simple and actionable feedback that invites interdisciplinary discussions, so that substantiated actions for improvement can be implemented. AF should not be seen as an isolated ad-hoc intervention, but as a recurrent, long-term, and organic improvement strategy that balances the primary aims of HCW (i.e. improving quality and safety of care for individual patients and HCW) and AMR-experts (i.e. reducing the burden of AMR).

**Conclusions:**

To unlock the learning and improvement potentials of audit and feedback, HCW’ and AMR-experts’ perspectives should be balanced throughout the whole AF-loop (incl. data collection, analysis, visualization, feedback and planning, implementing and monitoring actions). APM-AF should be flexible, so that both audit (incl. collecting and combining the right data in an efficient and transparent manner) and feedback (incl. persuasive and actionable feedback) can be tailored to the needs of various target groups. To balance HCW’ and AMR-experts’ perspectives a participatory holistic AF development approach is advocated.

## Background

Audit and feedback (AF) provides efficient and continuous opportunities to evaluate and improve the quality and safety of healthcare [1]. AF encourages practice changes by summarizing data about specific aspects of care (i.e. audit) and reporting the findings back (i.e. feedback) to healthcare workers (HCW). While AF is widely used, it yields modest and variable improvements in practice [2]. Therefore, many studies have focused on identifying key ingredients and understanding the working mechanisms of successful AF [3–5]. By now, we know that AF effectiveness depends on the targeted behaviour and the AF content, delivery, timing and context [2, 3, 5]. Known barriers to successful AF are a lack of feedback to HCW, feedback solely focused on what went wrong and a poor follow-up in terms of continuous quality improvement cycles [6–8]. Making sense of the audit data and translating data into actionable feedback is a challenge [9]. As a consequence, feedback often has little added value and HCW perceive AF merely as a tool to comply with external obligations (e.g. accountability to healthcare inspectorate) [6, 8]. Thereby, the potential of AF as an improvement and learning strategy is foregone.

Colquhoun et al. [5] further supported this by postulating that the limited effectiveness of AF might be caused by neglecting feedback-recipients in the AF development process. Literature on user-centred development has long addressed the importance of including end-users from the start of the development process [10, 11]. Involving end-users from the early stages of development and throughout the development process ensures that the AF is functional and useful, supporting the end-users’ goals, matching their working routines and fitting the organizational context [10]. Studies using a user-centred approach have shown positive results with behaviour changing interventions [12]. How this applies to audit and feedback strategies remains understudied. To examine how including end-users (in this case, HCW) in the early development process can improve AF strategies, this study focuses on a specific case where the potential added value of AF is large: preventing antimicrobial resistance (AMR) in hospitals.

Antimicrobial stewardship programmes (ASP), diagnostic stewardship programmes (DSP) and infection control programmes (ICP) are part of an integrated approach of AMR prevention measures (APM) that aim to reduce or prevent the increase in AMR [13]. Most APM activities directly interfere with HCW’ working routines. For example, by restricting the use of specific antibiotics or by checking prescriptions prospectively and advising to change if needed [14]. Concurrently, AMR-experts are concerned about deskilling HCW in APM as a result of restrictive interventions and an increased reliance on AMR-experts’ advice [15]. Therefore, studies focused on empowering HCW to take ownership of APM in their working routines are of added value for the integrated approach of APM. Audit and feedback does not interfere with HCW’ working routines, but provides objective insights in APM to support a reflective learning approach for HCW.

However, APM are currently developed and implemented in a top-down way [16, 17]. AMR-experts, such as clinical microbiologists, infectious disease specialists and infection control professionals develop and implement APM activities. HCW are hardly involved in APM development [18]. Therefore, HCW’ working routines and needs for APM support might not be sufficiently reflected, while HCW are responsible for integrating prescribed APM in their daily working routines while handling patients. AMR-experts on the other hand, also face challenges with APM, such as a lack of dedicated time, funding or personnel, competing high-priority initiatives, and opposition by HCW [19–21]. Promoting shared ownership of APM between HCW and AMR-experts is thus essential and could be realized by 1) improving awareness about (in) appropriate APM in HCW [22, 23], and by simultaneously 2) convincing AMR-experts of the relevance of including HCW in APM development.

To balance the top-down AF and expert-driven APM approaches with the bottom-up perspective of the HCW (or end-user), this study used a bottom-up participatory approach as a starting point for the development of AMR prevention measures audit and feedback (APM-AF). This paper focuses on the research question: **What are HCW’ needs and expectations for future APM-AF?** By answering this question, we aim to better match the APM-expert’s and HCW’ perspectives to optimize future AF strategies, thereby increasing the likelihood of APM-AF uptake and easier integration and use into practice [24].

## Methods

A qualitative semi-structured interview study was performed with HCW at a regional hospital in The Netherlands (687 beds) between December 2017 and March 2018. The University’s ethical committee approved this study (BCE18321).

### Study population and setting

Of the wide variety of HCW in the hospital, we focused on physicians, residents and nurses as key-stakeholders. Current AF strategies are focused on their work since they adopt APM into their daily working routines. Physicians, residents and nurses from the following departments were invited for an interview: Intensive Care, Emergency Department, Urology and Surgery. These departments handle vulnerable patients, which are often exposed to hospital admissions, invasive procedures and antibiotics, and thus are at increased risk of AMR [25].

In the regional hospital, microbiological diagnostics are locally organised and local guidelines on antibiotic prescribing are available in the form of online formularies. Expert consultations on medical microbiology, infectious diseases and hygiene are available by phone and in person.

Convenience sampling [26] was used by recruiting respondents through a previous hospital-wide survey [23]. Additionally, heads of departments were asked to invite HCW directly. No new interviews were planned when data saturation was achieved [27].

### Interviews

The interview scheme was developed by a multidisciplinary research team, including health scientists, psychologists, a clinical microbiologist and an infection control professional. Themes were based on results of our prior studies on HCW’ needs and expectations in APM support [23, 28, 29]. The interview scheme was tested with a physician and a nurse. The interview started with demographic questions and continued with questions about specific AF for APM themes:

- current AF strategies for APM (e.g. “How do you know the quality of your APM work?”, “Which feedback do you currently receive?”) and expectations for future AF strategies for APM (e.g. “How could AF support in improving APM?”);
- needs for future AF strategies for APM (e.g. “What would you like to know to determine your APM performance” and “How would you like to receive feedback on your APM performance?”);
- possible barriers or preconditions for successful AF (“Could you think of reasons or situations in which AF would not improve the quality of care?”).

Probing questions were asked to gain deeper insights in HCW’ experiences with current APM and expectations and needs for the content and delivery of APM-AF.

The interviews were conducted face-to-face at the hospital. Individual interviews were chosen over a collective focus group to reduce bias from social control due to respondents’ functions and specialties. During the interview, open-ended, broad questions were asked to obtain rich responses. The interviewer (JK) is an experienced interviewer with understanding of (the medical terminology of) AMR. After attaining informed consent, the interviews were recorded and transcribed verbatim.

### Data analysis

The transcribed interviews were coded in Atlas.ti (v8.2.30) by two researchers (JK and NBJ). Initial coding was deductive, based on the interview scheme themes as mentioned above. Within the themes sub-codes were created by open coding. Then, axial coding was performed to discover related concepts in the sub-codes. In this phase, variations within sub-codes were created if needed to explain differences within sub-codes. Analyst triangulation was applied (independent coding of 10% of the interviews by researcher NBJ) [30]. Disagreements between analysts mainly involved the use of different terminology for the sub-codes and variations. Differences were discussed until consensus was reached to increase internal validity [31].

## Results

### Respondents

Sociodemographic characteristics of the interview respondents (*n* = 16) are shown in Table 1. Respondents were physicians (*n* = 6), residents (*n =* 5) and nurses (*n =* 5). Respondents varied in age, function and experience in their function and experience at the hospital. Interviews took 45 min on average.

**Table 1:** Respondents characteristics

Respondents (*n* = 16)		
Age, mean (SD)	Years	41 (12,1)
Gender, n(%)	Male	8 (50)
	Female	8 (50)
Department, n(%)	Surgery	5 (31)
	Emergency Department	3 (19)
	Urology	5 (31)
	Intensive Care	3 (19)
Function, n(%)	Physician	6 (38)
	Resident	5 (31)
	Nurse	5 (31)
Function experience, mean (SD)	Years	11,1 (8,7)
Hospital experience, mean (SD)	Years	11,7 (12,9)

### Results interviews

Interrater reliability was found to be substantial (Kappa = 0.729, *p* < 0.001). For each interview theme various sub-codes and variations were constructed to represent the rich in-depth information that was retrieved in the interviews. The code schemes are presented below in Tables 2 and 3, including frequencies of sub-codes/variations and illustrative quotes. Sub-codes and variations are further elaborated upon below the tables.

**Table 2:** Needs for future APM-AF

Code	Sub-code	Variation	n	Quote
Needs audit	Content	Insights in diagnostics	6	“Do we use the right diagnostics for our patients? In other words, do we test too much or do we take the wrong tests?” P(17.36)
		Insights in empirical and targeted treatment	4	“I would like to know for a certain clinical presentation how we start our treatment, which antibiotics we start with.” P(13.29)
		Insights in infection control measures	4	“For infection control I would like to know what percentage gets clean clothes every day. And what effect that would have on the prevention of new infections. I would also like to know if hand hygiene is adequately applied and if people comply to the dress code. Also, the use of non-sterile or sterile gloves.” R(04.16)
		Insights in infection outcomes	3	“I would like to see how we perform in the hospital; how often do we have resistant micro-organisms and how often are these transmitted to other patients or personnel.” R(05.21)
		Insights in resistance patterns	5	“Insights in diagnostic results, resistance patterns, not for individual patients, but overall. How the resistance patterns have developed over time.” P(02.16)
	Norms	Benchmark	8	“If I would be compared to colleagues for example, that might be scary, but eventually you can learn a lot from it.” R(04.31)
		Trends over time	4	“You could do a baseline measurement, so how are we performing now. And then look how it evolves over time when you change things.” P(17.50)
Needs feedback	Content	Simple and concrete points of improvement and recommendations	7	“Some points we might be able to change ourselves, such as poor hygiene or so. But it may also be that policies need to be adapted, that certain antibiotics may or may not be given anymore. You really have to give something back that it is not just plain facts.” N(09.56)
		Feedback tailored to target group	8	“I would indeed stick to one group *[nurses or physicians]* and focus on that specific target group. Adapt the feedback to that group.” N(15.23)
		Substantiated recommendations	11	“I want to be convinced with good arguments. I understand that there are rules and you must adhere to them, so I adhere to them. But I find it very annoying when people can’t explain why. It seems logical and it is tangible, but if it is not scientifically proven, then I think you should thoroughly study it before you set a rule.” P(08.33)
	Form	Mail/ newsletter /poster	4	“I would like to receive some kind of newsletter online”. P(05.31)
		Interactive	13	“Just data is an empty shell. You have to present it, you have to discuss it, you have to work with it.” R(04.40)
	Frequency	Not too often, but recurrent	14	“Oh, not every week or month, then it is way too much. I think every six months, something like that. Because otherwise it will only overwhelm you and then it seems to be a goal and not a means for something.” P(17.62)
AF implementation	Approach	Positive	4	“I think positive reinforcement is better than focusing on the negative.” P(14.40)
		Transparent	1	“If there are consequences from AF, you have to explain in advance clearly why it happens with what purpose, that it is linked to a standard and that there is time to improve.” P(17.62)
	Ownership	Bottom-up	9	“It is also easier to hear feedback from someone you see more often than from someone who just shows up and has something to say about your work.” N(15.43)
		AMR/infection experts	8	“By someone who is knowledgeable about these topics.” N(09.49)
		Interdisciplinary	6	“It would be very valuable to have regularly multidisciplinary meeting with the bacteriologists and possibly infectiologists or an infection committee.” P(02.24)
		Supported by supervisors and management	3	“It must be supported by the organization, so people at the top, the management.” R(10.44)

*P* physician, *R* resident, *N* nurse

**Table 3:** Anticipated barriers and preconditions for future AF strategies for APM

Code	Sub-code		n	Quote
Anticipated barriers APM-AF	Difficulties with defining and operationalizing APM quality	Contradictive APM goals	11	“Quality for me means that the patient receives proper care”. R(04.05)
		APM quality determined by many aspects	4	“It is not only the person that needs to change, there might be other things. You need help from your colleagues, help from the environment; there are various sources that influence your behaviour”. (P17.64)
		Linking process and outcome indicators	4	“If someone has become septic after treatment at the department, that might not necessarily be wrong, but a natural course of an illness.” P(13.03)
	Difficulties with benchmarking		4	“That would also be good for departments, but then you would have to compare similar departments and that is difficult.” R(04.31)
	Information overload		7	“Because there is an overkill. There is so much information, you get feedback on too many things”. N(16.49)
	Registration burden		3	“For the quality it would be better if the doctor would not have to spend all the time on registering and controlling infection control measures, but if you want to do it properly, I suppose that is all in the game.” R(04.06)
	Measuring for the sake of measuring		5	“Look, a lot is being measured, but that does not necessarily lead to better care.” R(04.34)
Preconditions APM-AF	(Cost)-effectiveness of APM-interventions		8	“Costs also play a role, especially at this time. It should be cost-effective. Also, if it would require a lot of effort resulting in a relatively small result, then you really should consider the usefulness” P(05.59)
	Cultural safety		10	“Providing and receiving feedback is just difficult. You have to have a professional attitude”. P(17.58)

*P* physician, *R* resident, *N* nurse

### Current APM audit and feedback

HCW currently do not receive meaningful or actionable feedback on APM from audits to improve their own behaviour, nor to evaluate their working routines. Incidental audits by infection control professionals and workplace visits by the healthcare inspectorate result in general hospital-level feedback, while the audit content does not fully address the APM aims that HCW envision.

The feedback that is received, originates from direct interactions with AMR-experts and mainly focuses on ad-hoc decisions for individual patients. Medical microbiologists are easily consulted to check and adapt the planned diagnostics or treatment. Communication with infection control professionals was described as top-down (i.e. receiving instructions rather than consulting), which was deemed acceptable for unpopular, yet necessary decisions (e.g. commissioning isolated care for a specific patient, while there is a shortage of beds).

In sum, the most pressing challenges for APM-AF are:

- Audits on APM performance are limited;
- Audit content is expert driven and does not match HCW’ aims for APM;
- Feedback is not actionable for HCW;
- Finding a balance between top-down and bottom up.

HCW perceive the added value of AF for APM, because it will allow them to become more aware of AMR and of the contribution of their own behaviour and working routines to reduce the burden of AMR (i.e. reflective learning approach as opposed to ad-hoc decision-support for individual patients).

### Needs for future audit and feedback for APM

Table 2 presents HCW’ needs for future audit and feedback for APM. Needs are organized in needs for audit (content and norms), needs for feedback (content, form, frequency) and AF implementation (approach and ownership).

#### Needs audit

##### *Content*: Audits should cover both process- and outcome-measurements on DSP, ASP and ICP.

HCW were interested in audits on how many (quantity) and how well (quality) diagnostics were performed. They would also like audits to keep track of what empirical treatments are chosen for specific clinical presentations and if this was according to the local guidelines. After the start of an empirical treatment, antibiotics should possibly be adapted to match the results of diagnostic tests and HCW were interested to see how often such adjustments were actually made. Whereas the previous mentioned AF content focuses on process-measurements (direct reflections of HCW’ behaviour), HCW were also interested to see the outcomes (indirect and more uncertain reflections of HCW’ behaviour). They were interested in infection outcomes, such as prevalence figures of resistant micro-organisms and infections, and information about how often resistant micro-organisms are transmitted to other patients or staff. Lastly, HCW were interested in insights in overall local resistance patterns to see if the problem is indeed worsening and to check for possible needed adaptations of (empirical) treatment.

##### *Norms*: Audits should be mirrored to reference data.

Because stand-alone data is not meaningful, HCW would like to see their own performance data compared to some reference data. This could be a pre-agreed standard within the department or hospital, or it should allow for benchmarking between individuals, departments, similar hospitals or even regions. Another feasible alternative mentioned was to focus on trends over time to show progress. In this way, effects after APM-intervention implementation can also be evaluated.

#### Needs feedback

##### *Content*: Feedback should be simple, action-driven, tailored and substantiated.

Feedback should consist of more than plain data, since data are an “empty shell”. Data should be analysed and translated into simple and concrete points of improvements and recommendations. Feedback should be tailored to specific target groups (e.g. physicians or nurses), so that HCW feel that the feedback is relevant to them, unlike is often the case with current feedback.

Lastly, recommendations and planned APM-interventions should be substantiated, so that HCW know why measures are taken and what the expected effects are.

##### *Form*: Feedback should be embodied in interactive discussions.

HCW shared many possibilities of feedback forms. These range from informative mails, newsletters or posters to interactive presentations and education wherein the data and their implications can be extensively discussed. Two physicians suggested three additional forms of feedback: 1) analysing the data themselves (i.e. learning throughout the whole audit-feedback-cycle), 2) linking individual performance feedback to annual performance reviews and 3) direct feedback (on antibiotic prescriptions) in the form of a pop-up in the prescription system (i.e. decision-support).

##### *Frequency*: Feedback should be recurrent and requires long-term follow-up.

HCW did not want to receive feedback too often, because of the already intense information burden. Proposed feedback frequency varied from quarterly to yearly. HCW emphasized that AF should be recurrent and long-term to have impact, because changes in behaviour and culture take considerable time and effort. Long-term follow-up is also needed to measure the effects of APM-interventions. The preferred timing of feedback depended on its form (e.g. discussions during existing meetings).

#### Audit and feedback implementation

##### *Approach*: AF should positively and transparently reinforce HCW.

HCW indicated that a positive feedback approach (i.e. positive reinforcement) would work better than focusing on the negative (i.e. negative punishment). Positive reinforcement could for example be implemented by appraising high scores with rewards. If consequences were to be linked to AF, then transparency and clear instructions on the full AF procedure are required beforehand.

##### *Ownership*: AF should be organically shared throughout the organization.

Various opinions were raised about who should be responsible for the whole AF process. Some HCW indicated that it would be best to implement AF with HCW in the leading role, because imposing AF top-down would only lead to resistance. Receiving feedback from someone familiar on both a personal level (i.e. do I know the person) and on a work level (i.e. does the person know our work processes) is believed to increase the level of acceptance of feedback and therefore its effectiveness. Other possible AF owners were experts in the field of AMR and infections, because they have the required expertise and because they can serve as an objective outsider. However, a concern was that they might not always be aware of local working routines and might miss the clinical view that is required for the full treatment of the patient (not only the AMR focus). Therefore, AF should ideally be implemented in an interdisciplinary fashion, where AMR expertise and department/patient expertise are combined in the translation of data to feedback and APM-interventions. Lastly, HCW mentioned that the whole organization should support the AF initiative.

### Anticipated barriers and preconditions for future AF strategies for APM

Table 3 presents HCW’ anticipated barriers and preconditions for future audit and feedback strategies for APM.

#### Anticipated barriers for APM audit and feedback

##### Difficulties with defining and operationalizing APM quality.

HCW envisioned that a main challenge for auditing APM would be the fact that there is ambiguity about what APM quality entails. This could lead to discussions about valid measurements of APM quality. Several explanations for the ambiguity of APM quality were provided.

Firstly, ambiguity about the quality of APM is caused by contradictions in APM goals between HCW and AMR-experts. HCW indicated that they primarily aim their APM at providing the best possible care for individual patients. Few HCW mentioned preventing the spread of resistant micro-organisms or limiting AMR overall as a specific goal of their daily practice. Although they understand that AMR indeed is a threat to their patients and that this threat will likely increase in the future, providing the best care for their current patients seems to be more pressing. APM activities that aim to prevent the spread of resistant micro-organisms (e.g. treating patients in isolation) or to limit the overall AMR (e.g. awaiting test results to narrow AB treatment) are sometimes experienced as unfavourable for individual patients, while these are the activities that can be measured to define APM quality.

Second, HCW indicated that successful APM is determined by many aspects. Not only individual APM activities determine APM quality, but HCW are also depended on others in the full APM process (e.g. cultures taken by admission at another department) and on the context (e.g. availability of sufficient disinfectants or isolation rooms).

Third, concerns were raised about linking outcome indicators for APM (e.g. number of patients with resistant micro-organisms) to individual actions, because these outcomes are outside of HCW’ control (e.g. admitting patients carrying resistant micro-organisms or sepsis as a result of the course of a disease). As a result of the complexity of APM quality, not all APM measurements are expected to have impact on their approach to individual patients and thus the feedback provided would not lead to changes in behaviours.

##### Difficulties with benchmarking.

HCW also raised the concern that comparing data between regions, hospitals and departments would be difficult, because of differences in local resistance patterns and patient population. Comparing data on an individual level was mentioned as the most valuable for learning lessons, but concerns were raised about the availability of data.

##### Fear of registration and information burden.

HCW were afraid of more feedback on top of the feedback that is already provided (i.e. information overload) and of a registration burden that might come with AF. HCW described that many data are collected in healthcare, without necessarily improving the quality of care for individual patients (i.e. measuring for the sake of measuring). This belief is reinforced by the fact that HCW questioned the usefulness of some ICP guidelines. They feel like scientific evidence is limited and sometimes common sense neither urges compliance (e.g. clothing requirements). Measuring these aspects would have no added value to them.

#### Preconditions APM audit and feedback

##### Consider cost-effectiveness of AF follow-up.

An important precondition for the success of AF was the consideration of what can and should be done based on AF findings (i.e. AF follow-up). Cost-effectiveness of APM-interventions is therefore an important precondition for improvement suggestions. Concerns about costs were about both money (e.g. taking additional cultures) and effort (e.g. washing hands all the time).

##### Create an open and safe culture to discuss AF.

HCW emphasized that an open and safe culture in which you can address others’ behaviour is essential in improving APM or any other behaviour-related problem. Hierarchy sometimes hinders this open and safe culture. Especially residents and nurses explained that they would not address a medical specialists’ behaviour and some medical specialists acknowledged that they felt more comfortable with receiving feedback from peer specialists than from others.

## Discussion

This study revealed in-depth insights into HCW’ expectations and needs for future audit and feedback (AF) strategies for antimicrobial resistance prevention measures (APM). The following discussion reflects on this study’s findings, which results in specific recommendations for future (APM-)AF. To structure the discussion, we differentiate between reflections and recommendations on 1) audit and feedback itself (why, what, how) and 2) the development process of AF.

### Reflection on study findings

#### Audit and feedback: why?

Tracking and reporting are core elements for hospital APM as defined by the Centers for Disease Control and Prevention (CDC) [32]. However, this study showed that feedback in current AF strategies, if at all given, does not sufficiently support HCW in proactively considering AMR in their daily working routines. Therefore, our findings complement the CDC’s core elements, with the aim to evolve tracking and reporting into AF that serves both as a quality and safety strategy for the organization and as a learning system for HCW. By closely cooperating with HCW and AMR-experts from the start of the development process, we promote adopting an AMR-minded way of thinking and intrinsic motivation to take shared ownership needed for successful APM [33].

#### Audit and feedback: what?

An important finding of this study is the concern HCW raised about defining and operationalizing APM quality. This concern is not new to the field of quality improvement science [34, 35], yet not sufficiently discussed in the AMR-literature. A comprehensive discussion on the conceptual and operational definitions for AMR goes beyond the scope of this article, but we reflect upon three important considerations.

First, AF relies on the conceptual definition of what quality of healthcare, or in this case quality of APM, means [35]. This study illustrated that the definition of APM quality depends on whose perspective is incorporated. When looking at prior studies defining quality for APM [36–41], quality seems to be defined from a narrow AMR-perspective to fulfil the aims of evaluating stewardship programmes and benchmarking hospitals. Thereby aspects that define APM quality from the HCW’ perspective are not considered, meaning that the basics of AF do not optimally fit with HCW’ needs.

Second, closely related to conceptually defining quality is the operationalization of how quality is measured. Years ago, Donabedian introduced his conceptual model for the evaluation of quality of healthcare by measuring *structure*, *process* and *outcome* indicators [42]. More and more studies report on how indicators were selected in a systematic way [36–41], but far less studies have considered and scientifically tested the relationship between structure, process and outcome indicators to their clinimetric and psychometric properties [43]. Because of this the transparency, reliability and validity of the evidence-base for APM-interventions and strategies is diminished.

Lastly, and again closely related to beforementioned aspects of defining and operationalizing quality, is Donabedian’s notion that AF should be seen as an indicator rather than a definitive judgement of the quality of care [35]. The HCW’ needs we found in this study closely resonate with Donabedian’s reasoning: HCW need data-driven feedback as input for more objective discussions about their behaviour and working routines. They explicitly do not need feedback presented as judgement of their work, as currently is often the case.

Further translating these three key-considerations for audit and feedback in the APM-field, and any field for that matter, is a crucial step towards sense-making AF for quality improvement (and AMR reduction).

#### Audit and feedback: how?

Retrieving and analysing the data are crucial steps to unlock their potential. However, little scientific attention has been paid to translating data into actionable feedback and conveying the information to specific target groups [44]. We here highlight two study findings related to the translation and conveyance of actionable feedback. First, results of this study showed that HCW prefer positive feedback, while at the same time they acknowledge the need for top-down directions on unpopular but necessary decisions. This corresponds to findings of Fitzpatrick and Riordan, who studied the “carrot vs. stick” dilemma in the infection literature. They concluded that both top-down and bottom-up approaches are required for sustainable improvement [45]. Second, this study identified differences between physicians and residents on the one hand and nurses on the other hand. Whereas nurses expressed a clear need for practical “how-to” suggestions, physicians also showed interest in interactively discussing and even “playing-around” with the data themselves. Differences between nurses and physicians were also found in other studies within the context of AMR [46–49] and literature on AF also suggests other feedback recipient characteristics (e.g. intrinsic mastery goal orientation [5]) influence the success of AF. This calls for an AF system that can be tailored to fit the varying needs of various target audiences.

#### Developing AF (for APM)

From this study we have learned that incorporating a bottom-up approach reveals crucial aspects that are easily overlooked when following a more top-down expert-driven approach. More specifically, this study revealed gaps between different worlds on many levels:

- between healthcare workers and AMR-experts (e.g. bottom-up vs. top-down, individual needs vs. societal needs);
- between science and practice (e.g. balancing evidence-based with practice-based);
- between strict and flexible guideline implementation (e.g. pragmatic guideline implementation);
- between the fields of DSP, ASP and ICP (e.g. no integrated view on APM as reflected in the various literature sources required to highlight all aspects);
- between scientific disciplines (e.g. medical, behavioural, improvement, persuasive technology);
- between various databases (e.g. need for employing technical and data-science skills to extract and combine data from laboratory and hospital information systems).

### Improving future (APM) audit and feedback

From this study several lessons and recommendations can be drawn that need to be considered in the future development of audit and feedback strategies and more specifically in AF to support HCW in tackling the AMR-problem.

#### Improving audit and feedback: what?

Future studies should focus on how to balance different perspectives in defining quality of care by exploring differences in quality definitions by various stakeholders. For APM-AF, explicit attention should be paid to discuss the conceptual and operational definition of quality and to emphasize the added value for the patient, so that it better fits the HCW’ needs. To study this, research designs that explicitly consider evaluating multiple conflicting criteria in decision making (e.g. multi-criteria decision analysis [50]) and finding consensus (Delphi-studies [43]) are encouraged.

#### Improving audit and feedback: how?

Our study results underline the need for tailored information conveyance strategies for different target groups. This requires flexible AF, in which users can choose to what extent they would like to interact with various parts of the AF-cycle. Thereby, the required flexibility in the frequency of feedback can also be adjusted by HCW themselves, so that the information burden can be controlled. Furthermore, tensions between top-down and bottom-up approaches could be harmonized by adequately framing the feedback through smart and persuasive visualizations [51]. Here, we see a clear role for eHealth (e.g. interactive dashboards and eLearning), because it can tailor the data-representation and feedback to the demands of HCW and AMR-experts [52–54].

Furthermore, both HCW and AMR-experts are and always will be subjected to externally enforced directions. Therefore, the true challenge will be to explore when and how the gap between top-down and bottom-up should and can be bridged. Like many other studies, we emphasize that creating an open and safe culture is key in this process [55–57].

#### Future approach to developing AF (for APM)

To bridge the beforementioned gaps, future studies on the development and implementation of (APM-)AF should focus on incorporating evidence from literature in the fields of behaviour change [58], communication [59], data visualization [60, 61], persuasive technology [62] and data science [63]. Especially, attention should be paid to 1) collecting and combining the right data in an efficient and transparent manner (e.g. combining data from various IT-systems) and 2) communicating persuasive and actionable feedback (e.g. by using data visualizations). The CeHRes-roadmap [10] could guide the planning, coordination and execution of a participatory development process of data-driven APM-AF, since it fits well with the wicked AMR-problem because of its socio-technical and interdisciplinary-based participatory principles [64].

### Limitations

The exploratory and broad nature of this study has some limitations. First, the limited number and restricted specialities of HCW included in the interviews might raise concerns about the generalizability of the study findings. By combining findings from AF and APM literature with results from our previous studies and by including HCW from departments that have experience with AMR (i.e. they know what AMR means for their patients and work processes), we reached data saturation with the 16 interviews. Also, the findings provided us with a strong backbone for the analysis of the results. This resulted in themes that, combined with findings from other literature, are generalizable both within the fields of AF as APM. To validate the findings, future research could replicate this study with more and other HCW from other departments, hospitals or settings to determine in how far findings are context-specific.

Other limitations relate to the content of the interviews. One of our initial aims was to produce concrete recommendations for the design of AF for APM. However, after analysing the transcripts, we realized that during the interviews the question of when HCW would use AF in their daily practice was not sufficiently addressed. Because this question highly depends on the content (i.e. what is audited) and the form (i.e. how feedback is provided), this question should be addressed in a later stage of the development process. Also, we experienced that HCW found it difficult to express their needs, especially in terms of how feedback should be provided. Similar problems were reported by Crisan et al. [61], where participants showed clearer design preferences when asked to evaluate individual design elements than when evaluating entire reports. In future studies, mock-ups or prototypes could better guide the interview by supporting HCW to concretize and express their preferences. We have already started preparations for a next step in our research, in which this will be done.

Because this study operates on the crossroads between highly heterogenous fields, it was impossible to reflect upon all aspects of our findings. However, we believe that this paper contains important starting points for future research, both within and between the various involved fields. Therefore, this paper can be seen as an invitation to further discuss and crystallize audit and feedback, specifically for APM.

## Conclusion

Current audit and feedback strategies do not sufficiently support HCW in tackling the AMR problem. However, HCW belief that this could be realized via AF, because AF provides a call-for-action to tackle the AMR-problem through their daily working routines. HCW require insights into all facets of APM in their daily working routines (i.e. diagnostics, treatment and infection control). This should preferably be provided in the form of simple and actionable feedback that invites interdisciplinary discussions, so that substantiated actions for improvement can be implemented. AF should not be seen as an isolated intervention. Rather, it should be considered a recurrent, long-term, and organic improvement strategy that balances the aims of improving quality and safety of care for individual patients with reducing the burden of AMR. To realize sense-making AF, HCW’ and AMR-experts’ perspectives should be balanced throughout the whole AF-loop (incl. Data collection, analysis, visualization, feedback and planning, implementing and monitoring actions).

## Data Availability

The transcribed interviews are not publicly available due to privacy restrictions, but are available from the corresponding author on reasonable request.

## Abbreviations

AF: Audit and feedback
AMR: Antimicrobial resistance
APM: Antimicrobial resistance prevention measures
APM-AF: Antimicrobial resistance prevention measures audit and feedback
ASP: Antimicrobial stewardship programmes
DSP: Diagnostic stewardship programmes
HCW: Healthcare workers
ICP: Infection control programmes
